# Functional definition of the N450 event-related brain potential marker of conflict processing: a numerical stroop study

**DOI:** 10.1186/1471-2202-13-35

**Published:** 2012-03-27

**Authors:** Dénes Szűcs, Fruzsina Soltész

**Affiliations:** 1 Department of Experimental Psychology, Centre for Neuroscience, University of Cambridge, Cambridge CB2 3EU, UK

**Keywords:** Conflict processing, Interference, Subthreshold response activation, Stroop effect, Anterior cingulate cortex, Numerical distance effect, Numerical cognition, ERP, EEG

## Abstract

**Background:**

Several conflict processing studies aimed to dissociate neuroimaging phenomena related to stimulus and response conflict processing. However, previous studies typically did not include a paradigm-independent measure of either stimulus or response conflict. Here we have combined electro-myography (EMG) with event-related brain potentials (ERPs) in order to determine whether a particularly robust marker of conflict processing, the N450 ERP effect usually related to the activity of the Anterior Cingulate Cortex (ACC), is related to stimulus- or to response-conflict processing. EMG provided paradigm-independent measure of response conflict. In a numerical Stroop paradigm participants compared pairs of digits and pressed a button on the side where they saw the larger digit. 50% of digit-pairs were preceded by an effective cue which provided accurate information about the required response. 50% of trials were preceded by a neutral cue which did not communicate the side of response.

**Results:**

EMG showed that response conflict was significantly larger in neutrally than in effectively cued trials. The N450 was similar when response conflict was high and when it was low.

**Conclusions:**

We conclude that the N450 is related to stimulus or abstract, rather than to response conflict detection/resolution. Findings may enable timing ACC conflict effects.

## Background

Functional magnetic resonance imaging (fMRI) studies have identified brain structures involved in detecting/resolving conflict at the stimulus and response levels [1,2]. In addition, event-related brain potential (ERP) studies have identified a succession of events in stimulus/response conflict resolution [3-5]. However, so far studies have been limited by the fact that there is no independent measure of stimulus/response conflict. Hence, inferences were based on paradigmatic assumptions about the presence/absence of stimulus/response conflict in certain tasks. We have overcome this limitation [6] by measuring response conflict directly using electro-myography (EMG). Here we extended our previous methodology by manipulating response conflict at the paradigm level and checking the success of this manipulation by EMG. This method enabled us to determine whether a robust ERP marker of conflict detection/resolution, the N450 ERP wave, was related to stimulus or response conflict. The N450 has previously been linked to conflict effects emanating from the anterior cingulate cortex (ACC). Therefore, clarifying the functional significance of the N450 may enable us to time ACC conflict effects detected by fMRI.

In principle, Stroop conflict can appear at the level of stimulus representations (stimulus conflict) or at the level of motor response organization (response conflict). According to the stimulus conflict view, Stroop conflict appears because the representations of parallel processed stimulus dimensions are incongruent with each other [7]. According to the response conflict view, Stroop conflict appears during the organization of motor responses (e.g. vocalizations in oral or button presses in manual Stroop tasks). This so-called 'horse-race model' assumes that motor responses are primed by both task-relevant and task-irrelevant stimulus dimensions [8,9]. Conflict appears when parallel activated correct and incorrect responses begin to compete to dominate overt response activity. Discriminating between brain markers of stimulus and response conflict has been the subject of several neuroimaging studies.

fMRI studies have pointed to several brain areas involved in conflict processing. The most prominent of these areas is the ACC which has been shown to be active in nearly all studies examining conflict processing. According to a prominent theory, the conflict monitoring view [10-12], the activity of the ACC increases in response to conflicting information. Initially, the conflict monitoring view mostly emphasized the role of ACC in detecting/resolving response conflict [1,13,14]. However, evidence has now accumulated demonstrating that the ACC can be activated not only by response but also by stimulus conflict [2,15-18]. This suggests that the conflict-related activation of the ACC is wider than originally thought, and the ACC is involved in conflict monitoring across several information domains [10] Naturally, the ACC has not been the only brain area implicated in conflict processing. It has been shown that both the posterior parietal cortex [1,19] and the inferior parietal cortex [2] may be related to stimulus conflict processing. Similarly, response conflict has been shown to modulate premotor cortex [19] and dorsolateral prefrontal cortex activity [20].

ERP studies have identified a succession of events in paradigms involving conflict processing [4,6,21]. These studies have typically compared ERP amplitude in the incongruent vs. congruent conditions of various Stroop paradigms. The timing of these congruency-related effects ranges from relatively early (till about 220 ms after stimulus presentation) amplitude differences [21-23] to later (300-600 ms) amplitude effects [3-6,24]. Early effects have usually been interpreted as being related to stimulus conflict and late effects as being related to response conflict. The most prominent ERP effect is called the N450 effect. The N450 has been detected in most ERP studies of the Stroop conflict [3,5,6,24,25]. The N450 has negative polarity in incongruent minus congruent difference potentials, a latency of about 300-500 ms and centro-parietal topography. Previous ERP source localization attempts have consistently found that most variance in the topography of the N450 can be explained by dipoles in the ACC [3,6,26]. Henceforth, both the prominence of the N450 in conflict situations and source localization results linking the N450 to the ACC make it a likely conclusion that the N450 may be related to consistent conflict-related ACC activity detected by fMRI studies. However, a better understanding of the functional role of the N450 is hindered by the fact that it is unclear whether the N450 is related to stimulus or response conflict.

With regard to the above question, in some previous Stroop studies we measured the timing of response preparation by employing the Lateralized Readiness Potential (LRP), an ERP measure of correct/incorrect response activation [4,21,25,27]. In these studies the LRP index of incorrect response activation could potentially serve as a measure of response conflict. However, we were able to show an LRP marker of incorrect response activation, ie. response conflict, in correctly responded trials of normal adults only in one of four studies [25]. This was probably due to limitations of ERPs due to volume conduction (see [28,29] for details). In a recent study [6] we aimed to solve the above problem by measuring response conflict directly at the effector level by the synchronous recording of electro-myography (EMG) and electro-encephalography (EEG). Our approach was built on seminal studies using EMG in Flanker and Simon tasks [28,30-33]. We used a numerical Stroop paradigm where participants detected which of two Arabic digits was physically larger [34]. In the congruent condition the physically larger digit was also numerically larger than the other one (e.g. 2 8). In the incongruent condition the physically larger digit was numerically smaller than the other one (e.g. 2 8). EMG detected robust incorrect response hand activity in correctly responded trials of the incongruent condition but not of the congruent condition. This incorrect response hand activity temporally coincided with activity in the correct response hand (there was an overt correct response). This provides unequivocal evidence for the presence of response conflict independent of paradigmatic assumptions in Stroop tasks.^1 ^We also detected an N450 ERP effect in the incongruent, relative to the congruent, condition right after the offset of incorrect hand activity. This confirmed that the N450 is probably closely related to conflict detection/resolution [6]. However, because the focus of our previous study was the direct demonstration of response conflict in Stroop tasks (by comparing the incongruent and neutral/congruent conditions), we did not manipulate the amount of stimulus/response conflict within the incongruent condition. Therefore, we could not effectively test whether the N450 was related to stimulus or response conflict.

Here we have manipulated the amount of response conflict within the incongruent condition in order to determine whether the N450 ERP effect was related to stimulus or response conflict. In order to further generalize our previous findings to a slightly different task than previously used, we used the numerical decision task of the numerical Stroop paradigm. That is, participants decided which digit was numerically larger than the other one. In this task a behavioral response is given with the hand corresponding to the side (left vs. right) where participants see a larger number (Additional file 1: Figure S1). Response conflict was manipulated by applying a cue on a trial-by-trial basis. In 50% of trials numerical stimuli were preceded by an effective cue (an arrow pointing to the left or right) which pointed into the direction of the expected response by 100% accuracy. In 50% of trials numerical stimuli were preceded by a neutral cue (a horizontal line) which did not convey any information about the side of responding. Five percent of trials with effective and neutral cues were no-go catch trials where subjects were instructed to give no response. No-go trials served to force maintaining stimulus analysis in effectively cued trials. In order to further confirm that stimulus analysis happened in effectively cued trials, we also examined numerical and physical size distance effects (distance effects: accuracy and reaction time depends on numerical/physical size discrimination difficulty).

It was expected that response conflict will decrease in effectively cued trials relative to trials with neutral cues because the response can already be fully prepared in effectively cued trials by the time the stimulus appears. In contrast, we expected that stimulus analysis, hence, stimulus conflict, will be maintained in effectively cued trials. We used EMG to provide paradigm-independent direct evidence for the amount of response conflict in effectively and neutrally cued trials. That is, EMG was used in a novel way, to check the success of our response conflict manipulation. Our question was whether the N450 appears both when response conflict is high and when response conflict is low. If the N450 appears equally in both situations, then the N450 can be related to stimulus conflict detection/resolution. In contrast, if the N450 is more expressed when response conflict is high, relative to when it is low, then it is more likely that the N450 is related to response conflict detection/resolution. We also used the peak latency of the P300 ERP wave because several previous Stroop studies [27,35,36] used this as a marker of stimulus conflict. However, it is to note that while this view is suggested by some [37], it is debated by others [38,39].

## Methods

### Participants

20 adults were tested. 3 participants were excluded from the sample because of EEG artifacts. Two more participants were excluded because they did not follow instructions closely (see Results). Therefore, 15 adults' data (mean age 23 ± 0.4 years; 5 males) were analyzed. Participants were graduate and undergraduate students at the University of Cambridge. The study received ethical approval from the Psychology Research Ethics Committee of the University of Cambridge.

### Stimuli and procedure

A stimulus consisted of two Arabic digits shown simultaneously in the middle of a 19-inch computer screen. Stimuli were white characters presented on black background. A trial started with a cue shown for 800 ms. This was followed by a delay for about 1000 ms (a random interval between ± 50 ms was added to 1000 ms in order to suppress alpha activity which may time-lock to stimulus-presentation). Than two Arabic digits appeared on the left and right of the fixation cross for 800 ms. The inter-trial interval was 1000 ms.

The following number pairs were used as stimuli in the congruent and incongruent conditions: 1-2, 2-1, 8-9, 9-8, 1-8, 8-1, 2-9 and 9-2. The following number pairs were used in the neutral condition: 1-1, 2-2, 8-8 and 9-9. The two digits in a pair were of different physical font size. In one half of trials the physical size of digits was 40 and 45 points. In the other half the physical size of digits was 40 and 50 points. This manipulation defined the task difficulty factor: the physical size difference between digits was either small (5 points: difficult condition) or large (10 points: easy condition). The numerical distance between digits was either 1 or 7 in the congruent and incongruent conditions (numerical distance factor), and 0 in the neutral condition. Exactly the same digits were used for both the numerical distance 1 and 7 conditions. By using two numerical distances we kept the number of levels of the task-irrelevant factor at the same value of the number of levels of the task-relevant factor. In the congruent condition the physically larger digit was also numerically larger than the other one. In the incongruent condition the physically larger digit was numerically smaller than the other one. In the neutral condition the two digits were of the same numerical value. In half of the trials the physically larger number appeared on the right, in the other half, on the left. The same held for the position of the numerically larger number as well. Congruency, size difference, numerical distance, and the side of the response (left or right hand) were manipulated orthogonally.

Participants' task was to indicate with a button press whether the numerically larger number appeared on the left or on the right. Participants pressed response buttons with their thumbs. Half of the trials in each possible condition were preceded by an effective cue, the other half by a neutral cue. The effective cue was the drawing of a horizontal arrow which could point either to the left or to the right. Participants were told that effective cues predicted with 100% accuracy whether they would have to respond to the numerical stimuli with the left or with the right hand (depending on whether the arrow pointed to the left or to the right). The neutral cue was a horizontal line. Participants were told that neutral cues did not provide information on the required response. 5% of trials with both an effective and neutral cue were no-go catch trials. In no-go trials the digits were replaced by hyphen signs. Participants were instructed not to give any behavioral response in catch trials. Catch trials served to make sure that participants withhold their response till digits appear, and to force maintaining stimulus analysis in effectively cued trials. Catch trials were distributed equally across all conditions.

Numerical stimuli in stimulus sequences were pseudo-randomized in a way that controlled for the number and distribution of response side (left or right) combined with congruency in one stimulus sequence. All 36 possible combinations of response side (4 combinations: right after right, left after right, left after left and right after left) and congruency (9 possible pairs of congruent, incongruent and neutral) were controlled for and evenly distributed within a stimulus sequence in order to avoid any response preparation bias. Each participant received a unique pseudo-randomized sequence of stimuli, equating response preparation effects both within- and across subjects. There were 9 experimental blocks with 96 trials in each block (864 stimuli). The experiment was preceded by 48 practice trials. Stimuli were delivered by Presentation 11 (Neuro-behavioral systems).

### Behavioral data analysis

In order to reject fast guesses only trials with RT longer than 150 ms were accepted for analysis. Accuracy and RT were analyzed by Cue (neutral vs. effective) × Congruency (congruent, incongruent and neutral) × Numerical Disance (small vs. large) × Physical Distance (small vs. large) repeated measures ANOVAs. Tukey-HSD tests were used for post-hoc analyses. In a second analysis congruent minus neutral, incongruent minus neutral, and incongruent minus congruent difference accuracy and RT values were computed. This data was also analyzed by Cue × Congruency repeated measures ANOVAs. Contrasts between statistical cells were examined by Tukey-HSD tests in both ANOVAs. In all behavioral and physiological ANOVAs Greenhouse-Geisser epsilon (ε) correction was used when necessary. Original df values and corrected p values are reported. Behavioral data was analyzed in Statistica 7.0.

### Electro-myography (EMG) recording, pre-processing and analysis

EMG was measured by EMG110C amplifiers using an MP150 data acquisition unit (Biopac Inc.). Two disposable cloth-based hypoallergenic Ag-AgCl EL504 recording disc electrodes were connected by 110S shielded touch-proof leads. Active electrodes were placed along the left and right flexors of the thumb (flexor pollicis brevis). An electrode on the left elbow served as ground. Before electrode application the skin was washed with soap, gently abraded and washed with alcohol. The electrodes were attached by adhesive solid gel. EMG was sampled at 1000 Hz, band-pass filtered between 10-250 Hz [40], rectified and scaled relative to the maximum amplitude measured in each response hand in each individual [41]. Hence, EMG is expressed as percent of the maximum value measured. EMG was also baseline-corrected relative to the -100 to 0 ms interval preceding stimulus presentation. EMG epochs extended from -100 to 998 ms relative to stimulus presentation.

EMG data was analyzed in Matlab 7.1 and Statistica 7.0. First, individual EMG was smoothed by a 50-ms-wide running average window. Second, the deviation of EMG amplitude from zero was tested by point-by-point one-sample t-tests run against zero for each Cue × Congruency condition. Significant deviations from the pre-stimulus baseline were considered a sign of significant motor activation. Henceforth, significant deviations will be called "EMG activations". Deviations from zero were considered significant if they reached significance over a minimum of 20 consecutive sampling points at p < 0.025. In a third step Cue × Congruency repeated-measures ANOVA was run on the mean EMG amplitude of intervals found to show significant deviations from zero.

### Event-related brain potential recording and pre-processing

EEG was recorded by an Electrical Geodesics system with a 129-channel Hydro-Cell Net. Electrode positions are shown in Additional file 2: Figure S2. The sampling rate was 500 Hz, an on-line band-pass filter of 0.01-70 Hz was used. The data was band-pass filtered between 0.01-30 Hz offline, and was recomputed to average reference. Epochs extended from -100 to 998 ms relative to stimulus presentation. Data was baseline corrected relative to the -100 to 0 ms interval. Epochs containing voltage deviations exceeding ± 100 μV relative to baseline at any of the recording electrodes and epochs containing ocular artifacts (visually detected by the experimenters at electrodes below, above and next to the eyes) were rejected. Analyses were run on both stimulus-locked and response-locked data. Both stimulus-locked and response-locked data was baseline corrected according to a common -100 to 0 msec prestimulus baseline.

### Event-related potential analysis

The overall temporal course of congruency effects was illustrated by the global field power (GFP). The GFP is computed as the mean potential deviation of all recording electrodes, and it reflects the spatial standard deviation of the data [42,43]. A large GFP is computed when ERPs show high peaks and troughs and steep potential gradients simultaneously on several electrode channels. Hence, the GFP is an excellent method for summarizing robust ERP effects appearing at many electrodes in a single curve. Importantly, the GFP characterizes the latency of robust distributed ERP effects by a single curve.

Effects in ERP amplitude were first examined by point-by-point Cue × Congruency × repeated-measures ANOVAs. In order to protect against Type-I errors a conservative significance level of *p *< 0.005 was chosen for EEG analysis. Time intervals where statistical effects reached significance (*p *< 0.005) over a minimum of 10 consecutive sampling points at least at 6 electrode channels were considered to demonstrate significant effects. Cue × Congruency ANOVAs were run on the average mean amplitude of electrodes demonstrating significant effects according to point-by-point ANOVAs (the mean amplitudes were computed for time intervals with significant effects). The topography of congruency effects was visualized as congruent minus neutral, incongruent minus neutral, and incongruent minus congruent difference potentials.

An analysis compared the topography of the N450 latency across effectively and neutrally cued conditions. The mean amplitude of the N450 was determined between 280-420 ms at the vertex electrode (electrode 129) and at electrodes in the innermost three electrode circles of the sensor net (altogether 31 electrodes). A Cue × Electrode ANOVA was run on incongruent minus neutral difference potentials. Another Cue × Electrode ANOVA was run on incongruent minus congruent difference potentials. A further analysis compared the peak latency of the N450 across effectively cued and neutrally cued conditions. Peak latencies were determined for incongruent minus congruent and incongruent minus neutral difference potentials. The peak latency of the N450 was defined as the sampling point with the most negative amplitude between 250-550 ms. Latency values were measured at the vertex electrode (electrode 129) and at electrodes in the innermost three electrode circles of the sensor net (altogether 31 electrodes). Cue × Electrode ANOVAs compared peak latencies for both incongruent minus congruent and incongruent minus neutral difference potentials.

The peak latency of the P300 wave was determined between 300-700 ms. The peak latency was defined as the sampling point with the most positive amplitude on 14 centro-parietal electrodes (electrodes 7, 129, 106, 31, 80, 54, 55, 79, 61, 62, 78, 67, 72, and 77). These electrodes were chosen because the maximum amplitude of the P300 happened at these electrodes. The peak latency of the occipital P100 wave (sampling point with the most positive amplitude) was determined between 70-150 ms. The peak latency of the occipital N200 wave (sampling point with the most negative amplitude) was determined between 80-220 ms. The peak latency of the P100 and N200 was measured on electrodes 65, 66, 70, 68, 69, 73, 83, 84, 90, 88, 89 and 94 where these waves showed the largest amplitude. The peak latency of ERP waves was tested by Cue × Congruency × Electrode repeated-measures ANOVAs.

In order to compare a central measure of response activation to EMG data, we also computed the Lateralized Readiness Potential (LRP). The LRP was computed as proposed by Gratton et al. [28]:

$$\left[{\left(\text{ER}-\text{EL}\right)}_{\text{LEFT}\;\text{HAND}\;\text{response}}+{\left(\text{EL}-\text{ER}\right)}_{\text{RIGHT}\;\text{HAND}\;\text{response}}\right]/2,$$

where EL denotes the amplitude of the ERP at an electrode placed over the left motor cortex, and ER denotes the amplitude of the ERP at an electrode placed over the right motor cortex. In the traditional 10-20 electrode system electrode C3 is used as EL and electrode C4 is used as ER. Hydro-Cell Net electrode 36 has equivalent position to electrode C3 and Hydro-Cell Net electrode 104 has equivalent position to C4. Hence, electrode 36 was used as EL, and electrode 104 was used as ER. According to convention a negative LRP indicates a correct response tendency, and a positive LRP indicates an incorrect response tendency. The deviation of the LRP from baseline was tested by point-by-point two-tailed one-sample t-tests run against zero. Effects were considered significant when they reached significance at *p *< 0.025 over a minimum of 10 consecutive sampling points (20 ms).

## Results and statistical analyses

### Behavioral results

First, the validity of the go/no-go instruction was checked. Two participants pressed any of the response buttons during more than 50% of the effectively cued no-go trials (during 14 and 20 trials). These participants were excluded from the sample because they did not follow instructions effectively. The fifteen participants remaining in the sample gave 2-8 (mean and standard error: 5.35 ± 1.3) incorrect responses in the effectively cued no-go condition (mean RT and standard error: 328 ± 6 ms) and a single participant gave a single incorrect response (RT: 136 ms) in the non-cued no-go condition. The above suggests that most participants could withhold the response effectively till the stimuli appeared and that the cue manipulation had a robust effect on the motor preparation of the participants.

**Figure 1:**
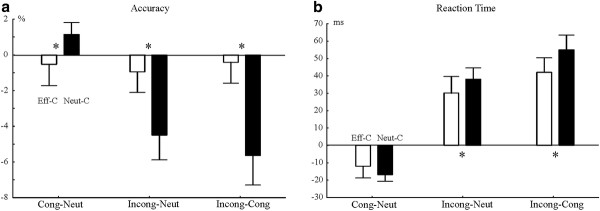
**Accuracy (A) and reaction time (B) difference values**. Stars (*) show when pair-wise contrasts between the effectively (Eff-C) and neutrally cued (Neut-C) conditions were significantly different. X axis: Cong-Neut: Congruent-Neutral; Incong-Neut: Incongruent-Neutral; Incong-cong: Incongruent-Congruent. 95% confidence intervals are shown.

Accuracy and RT in Cue × Congruency conditions are shown in Table 1. Difference accuracy values are shown in Figure 1A. Accuracy data was analyzed after excluding potential fast guesses responded faster than 150 ms (0.6% of data). Effectively cued trials were responded to less accurately than neutrally cued trials (correct: 94.7% vs. 97% ms; F(1,14) = 43.36; *p *< 0.0001). In accuracy, there was a Congruency effect (F(2,28) = 23.94; ε = 0.642; *p *< 0.0001), and a Cue × Congruency interaction (F(2,28) = 21.98; ε = 0.721; *p *< 0.0001). Post-hoc Cue × Congruency Tukey contrasts revealed that effectively cued trials were responded to at the same level of accuracy in all congruency conditions. In contrast, accuracy differed between all congruency levels in neutrally cued trials (*p *< 0.0001). There was a main effect of numerical distance (F(1,14) = 10.62; *p *< 0.005; small vs. large distance: 95.0 vs. 96.5%) and physical size distance (F(1,14) = 5.91; *p *= 0.028). There was no Cue × Numerical distance interaction (*p *= 0.4). However, there was Cue × Physical size distance interaction (F(1,14) = 20.63; *p *< 0.0001). The interaction appeared because there was a physical size distance effect in the neutrally cued condition (97.8% vs. 95.9%; Tukey *p *= 0.002) but not in the effectively cued condition (94.5% vs. 94.9%; *p *> 0.14).

**Table 1:** Reaction Time (ms), Accuracy (%) and P300 peak latency (ms).

	Cued			Non-Cued		
	
	Congruent	Incongruent	Neutral	Congruent	Incongruent	Neutral
**Reaction Time**	420	462	432	474	529	491
	10	12	11	9	11	10
**Accuracy**	94.2	93.8	94.7	99.2	93.6	98.1
	0.4	0.6	0.6	0.4	1.1	0.5
**P300 latency**	412	459	430	418	445	431
	38	34	38	43	53	41

Upper rows contain means, lower rows contain standard errors

First, original RTs were examined. Effectively cued trials were responded 61 ms faster than neutrally cued trials (437 vs. 498 ms; F(1,14) = 105.48; *p *< 0.0001). There was a Congruency effect (F(2,28) = 130.65; ε = 0.801; *p *< 0.0001), and a Cue × Congruency interaction (F(2,28) = 8.20; ε = 0.871; *p *= 0.0026). Post-hoc Cue × Congruency Tukey tests signaled that all possible Cue × Congruency contrasts were significantly different from each other (0.0001 <*p *< 0.0003). That is, there were congruency effects in both effectively and neutrally cued trials. Difference RTs are shown in Figure 1B. Cue × Congruency Tukey contrasts in difference RTs showed that the incongruent-neutral (*p *= 0.0381; effectively cued: 30 ms; neutrally cued: 38 ms) and incongruent-congruent (*p *= 0.0003; effectively cued: 42 ms; neutrally cued: 55 ms) difference values significantly differed when comparing effectively cued and neutrally cued trials. The congruent-neutral difference values did not differ across effectively cued and neutrally cued trials (effectively cued: -12 ms; neutrally cued: -17 ms).

In original RTs there were main effects of numerical distance (F(1,14) = 70.28; *p *< 0.0001) and physical size distance (F(1,14) = 25.33; *p *< 0.0002). Even when examined by multiple-testing corrected post-hoc Tukey tests, the numerical and the physical distance effects appeared significantly both in the effectively cued and in the neutrally cued trials (Numerical distance effects [small distance vs. large distance]: effectively cued condition: 449 vs 427 ms [*p *< 0.0002]; neutrally cued condition: 511 vs. 486 ms [*p *< 0.0002]. Physical size distance effects: effectively cued condition: 435 vs 441 ms [Tukey *p *< 0.07; Fisher-LSD *p *< 0.02]; neutrally cued condition: 493 vs. 503 ms [*p *< 0.0002].). There were no Cue × Numerical distance (*p *> 0.5) or Cue × Physical distance (*p *> 0.3) interactions. The presence of numerical and physical size distance effects in the effectively cued condition shows that stimuli were analyzed in this condition as well.

### Electro-myography

**Figure 2:**
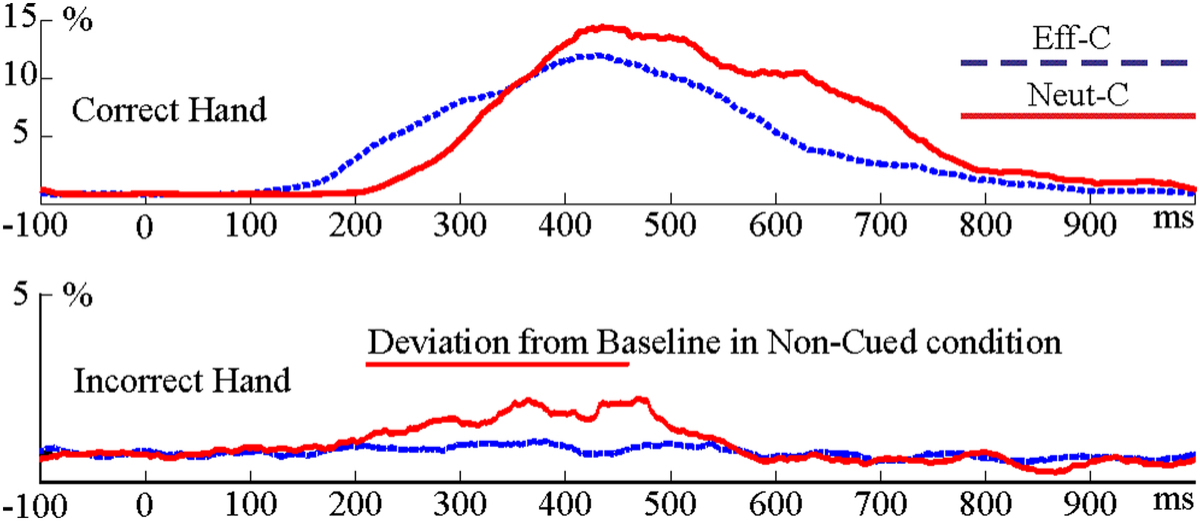
**EMG signal in the correct and incorrect response hand in the incongruent condition in the effectively (Eff-C) and neutrally cued (Neut-C) conditions**.

**Figure 3:**
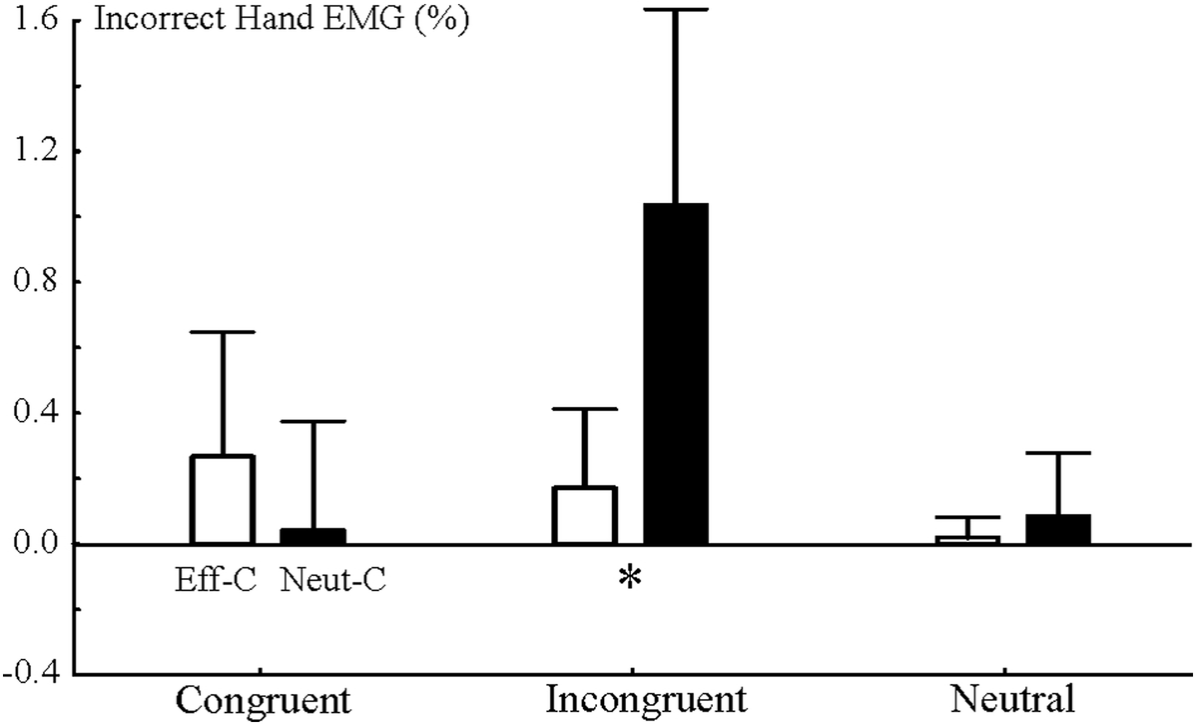
**The amplitude of the EMG signal in the incorrect hand in the effectively (Eff-C) and neutrally cued (Neut-C) conditions**. 95% confidence intervals are shown.

The EMG of the correct and incorrect response hands in the incongruent condition is shown in Figure 2. According to point-by-point tests the EMG of the incorrect hand showed significant activation exclusively in the neutrally cued incongruent condition between 210-453 ms. The mean EMG amplitude in the incorrect hand between 210-453 ms is shown in Figure 3. An ANOVA was run on this mean EMG amplitude. There was a Congruency effect (F(2,28) = 8.94; ε = 0.873; p = 0.0017) and a Cue × Congruency (F(2,28) = 8.94; ε = 0.693; *p *= 0.0040) interaction. Cue × Congruency Tukey contrasts showed that incorrect hand EMG activity was larger in the neutrally cued incongruent condition than in any other conditions (all contrasts: *p *< 0.0043). Other cells did not differ from each other (all contrasts: *p *> 0.7). The EMG of the correct hand significantly deviated from the baseline in all Cue × Congruency conditions (see Additonal file 3: Figure S3).

### Event-related brain potentials

**Figure 4:**
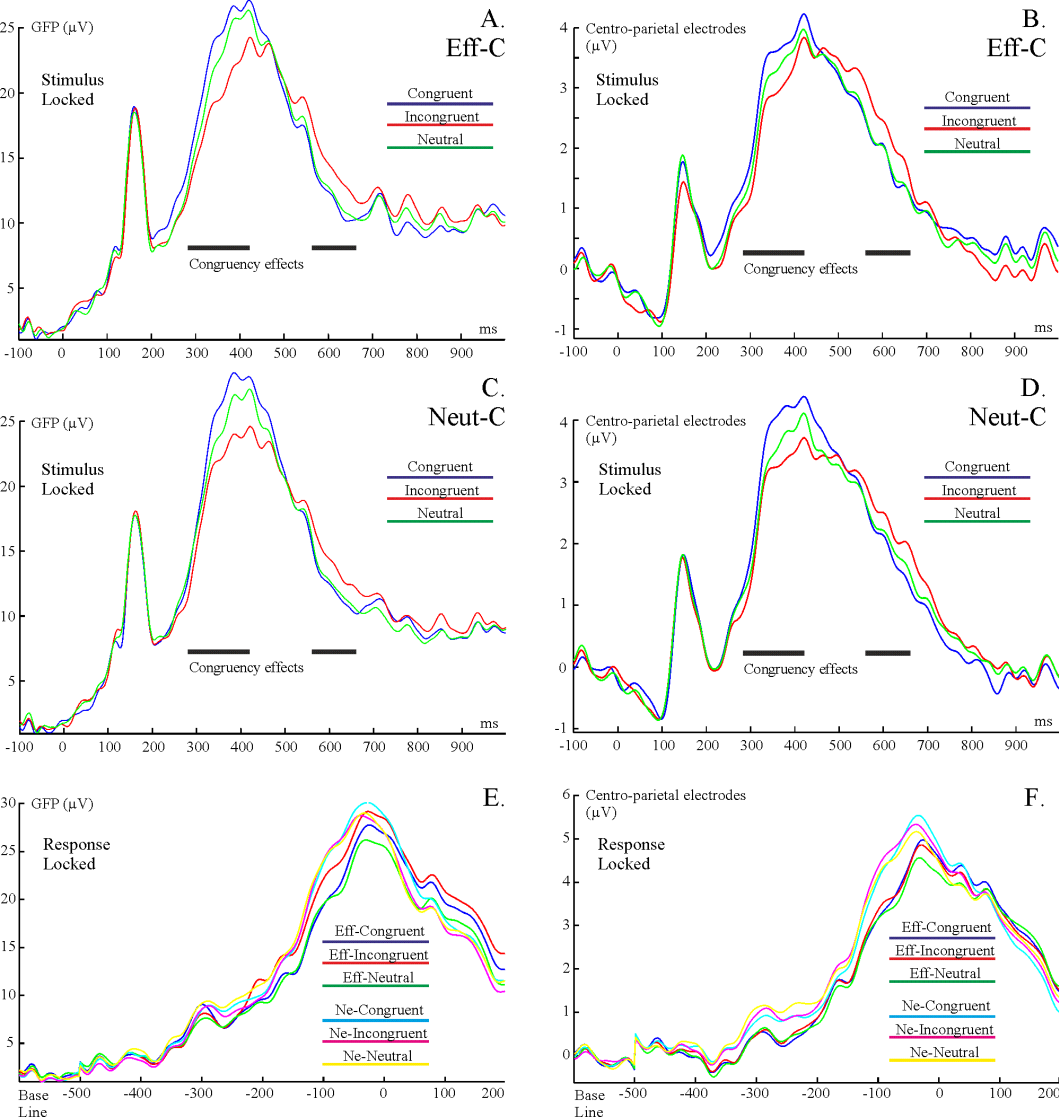
**ERPs in the effectively (Eff-C) and neutrally cued (Neut-C) conditions**. The time course of congruency effects detected by ANOVAs are shown by thick horizontal lines. (A-D) Stimulus-locked averages. (A) Global field power (GFP) in the effectively cued condition. (B) ERPs in the effectively cued condition. (C) GFP in the neutrally cued condition. (D) ERPs in the neutrally cued condition. (E) GFP in all Cue × Congruency conditions in response locked averages. (F) ERPs in all Cue × Congruency conditions in response locked averages.

**Figure 5:**
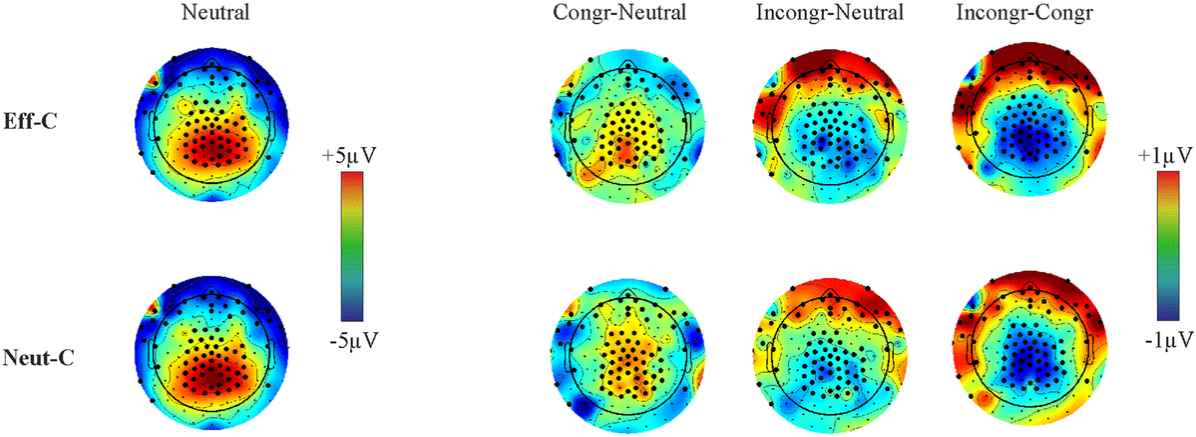
**The topography of congruency effects (p < 0.005) between 280-420 ms (the N450 effect) in the effectively (Eff-C) and neutrally cued (Neut-C) conditions**. The amplitude of ERPs in the neutral condition is shown on the left for reference. The amplitude of difference topographies is shown on the right (Congr: Congruent; Incongr: Incongruent). The upper row shows the effectively cued condition, the lower row shows the neutrally cued condition.

**Figure 6:**
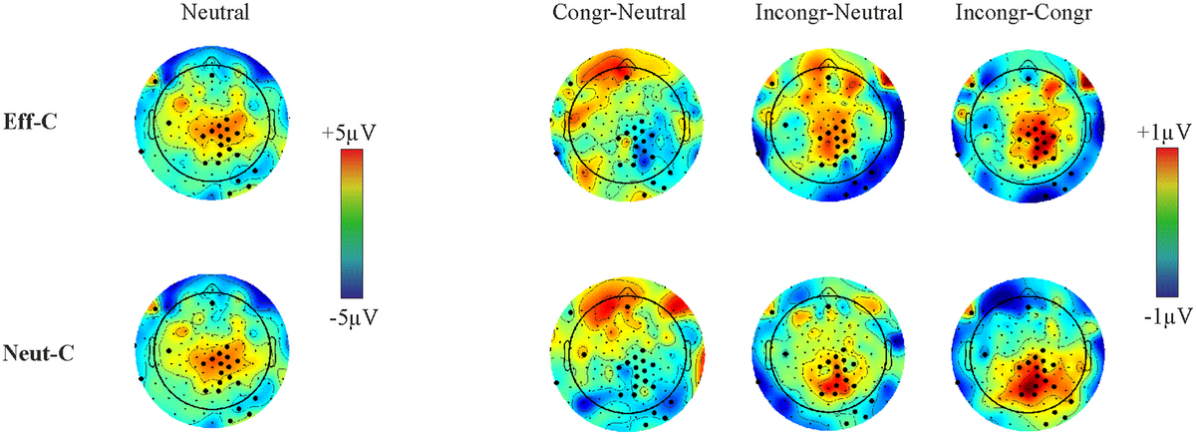
**The topography of congruency effects (p < 0.005) between 560-660 ms in the effectively (Eff-C) and neutrally cued (Neut-C) conditions**. The amplitude of ERPs in the neutral condition is shown on the left for reference. The amplitude of difference topographies is shown on the right (Congr: Congruent; Incongr: Incongruent). The upper row shows the effectively cued condition, the lower row shows the neutrally cued condition.

**Figure 7:**
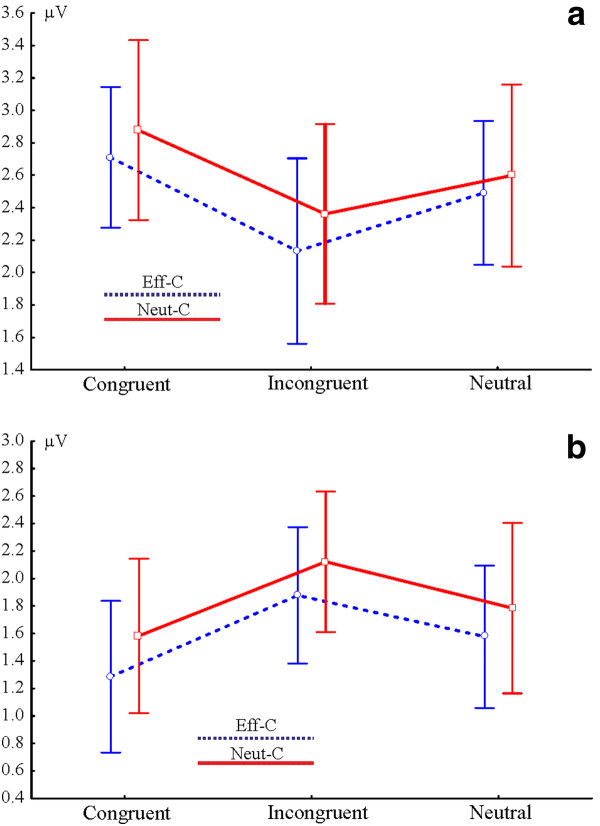
**Congruency effects in ERP amplitude in the effectively (Eff-C) and neutrally cued (Neut-C) conditions**. (A) ERP amplitude of the N450 effect (280-420 ms). (B) ERP amplitude of the congruency effect between 560-660 ms.

Grand-average ERPs averaged for representative centro-parietal electrodes (electrodes 129, 21, 55, 80, 54, 55, 79, 61, 62 and 78) as well as the GFP are shown in Figure 4 and in Additonal file 4: Figure S4. The amplitude of ERPs was examined by point-by-point Cue × Congruency ANOVAs. There were no Congruency effects or Cue × Congruency interactions in response locked data (Figure 4E-F). Hence, all the remainder of results refers to stimulus locked data. In stimulus-locked data no reliable Cue effects and Cue × Congruency interactions were found at the original alpha level of *p *< 0.005. In contrast, ANOVAs identified a main effect of congruency in two time windows (*p *< 0.005). Appropriate scalp topographies and electrodes with significant effects are shown in Figures 5 and 6. The first congruency effect appeared over several centro-parietal electrodes between 280-420 ms (Figure 5). According to its timing, topography and polarity we identify this effect as the N450. The mean amplitude of the N450 was determined at all centro-parietal electrodes demonstrating significant congruency effects. The average amplitude of all electrodes is shown in Figure 7A. This average amplitude was entered into a Cue × Congruency ANOVA. There was a congruency effect (F(2,28) = 19.02; ε = 0.998; *p *< 0.0001) and there was no Cue effect or Cue × Congruency interaction (F < 1; p > 0.7). According to post-hoc Congruency Tukey contrasts the incongruent vs. neutral (*p *= 0.0063) the incongruent vs. congruent (*p *= 0.0001) and the congruent vs. neutral (*p *= 0.0241) amplitude differences were significant. A sidenote is that in Figure 4 visible amplitude deviations between 420-500 ms resemble potential congruency effects. These amplitude deviations were not significant according to point by point tests. Nevertheless, we confirmed this by running an additional ANOVA on the mean amplitude of ERPs between 420-500 ms. There were no significant effects (all: *p *> 0.19). The comparison of N450 topography across effectively and neutrally cued conditions showed no Cue × Electrode interactions in difference potentials (incongruent-neutral: *p *> 0.96 and incongruent-congruent: *p *> 0.98). That is, we conclude that the same N450 effect appeared in both the effectively and neutrally cued conditions. Similarly, the latency of the N450 was compared across effectively cued and neutrally cued conditions. There were no significant effects on N450 latency.

The second congruency effect appeared between 560-660 ms (see Figure 6). The mean amplitude of the effect was determined at all centro-parietal electrodes demonstrating significant congruency effects (see amplitude in Figure 7B). The mean amplitude of the effect was tested by a Cue × Congruency ANOVA. There was a congruency effect (F(2,28) = 13.73; ε = 0.921; *p *= 0.0001) and there was no Cue effect or Cue × Congruency interaction (F < 1; *p *> 0.9). According to post-hoc Tukey congruency contrasts the incongruent vs. neutral (*p *= 0.0172) and the incongruent vs. congruent (*p *= 0.0002) amplitude differences were significant.

In order to avoid Type-II errors, we re-ran Cue × Congruency ANOVAs with a lower, *p *< 0.025, statistical threshold. These ANOVAs identified Cue effects between 60-100, 320-380 and 530-600 ms (see Additional file 5: Figure S5). Cue × Congruency interactions were identified between 230-280 ms (see Additional file 6: Figure S6). Importantly, there was no sign of Cue × Congruency interactions during the time-range of the N450. There were no such interactions even when the statistical threshold was set to *p *< 0.05.

The P300 latency is shown in Table 1. There was a congruency effect on P300 latency (F(2,28) = 25.33; ε = 0.885; *p *< 0.0001). Post-hoc Congruency Tukey contrasts showed that all congruency levels differed from each other. The P300 peaked 37 ms later in incongruent than in congruent trials (Tukey p = 0.0001), it peaked 21 ms later in incongruent than in neutral trials (Tukey *p *= 0.0011), and it peaked 15 ms earlier in congruent than in neutral trials (Tukey *p *= 0.0139). There was no Cue × Congruency interaction in P300 latency. Again, as the P300 peak latency is frequently used as a measure of stimulus analysis speed, this suggests that stimulus analysis happened in both the effectively and neutrally cued conditions. There was a congruency effect in P300 amplitude (F(2,28) = 6.99; ε = 0.713; *p *= 0.0091). The P300 amplitude was more negative in the incongruent than in the congruent condition (4.95 vs. 5.41 μV; Tukey *p *= 0.0026). There were no significant main effects or interactions on the peak latency and peak amplitude of the occipito-parietal P100 and N200 waves.

The LRP was examined as a central measure of response activation. The LRP is shown in Additional file 7: Figure S7. The LRP showed significant negative deflections (correct response activation) during the response period in both the effectively and neutrally cued conditions. However, the LRP showed no sign of reliable positive deflections (incorrect response activation) before and during the response period in neither the effectively cued nor the neutrally cued condition.

## Discussion

We have combined ERPs and EMG in order to determine whether a robust ERP signal of conflict processing, the N450 effect [3,5,6,24-26], is related to stimulus or response conflict. We have manipulated response conflict on a trial-by-trial basis by showing an effective or a neutral cue before the Stroop stimuli. Using a novel approach, EMG served to validate the manipulation of the amount of response conflict in a direct manner, independent of paradigmatic assumptions [6]. We have confirmed that response conflict was indeed higher in the neutrally, than in the effectively, cued condition. At the same time there was clear evidence that stimulus processing was maintained in both the neutrally and effectively cued trials. The N450 effect appeared only in stimulus-locked but not in response-locked trials. Overall, we conclude that remaining conflict effects in the effectively cued condition can be attributed to stimulus conflict. Data suggest that the N450, probably an ACC-related ERP marker of conflict, is not related to response conflict. Rather, it is either related to the processing of stimulus conflict or to the processing of an abstract level of conflict.

### Cueing and response conflict

EMG has confirmed that the cueing manipulation worked as expected. There was a highly significant difference in the amount of incorrect response activation between the neutrally and effectively cued conditions, incorrect response activation being much larger in the neutrally than in the effectively cued condition. That is, as expected, response conflict was considerably higher in the neutrally than in the effectively cued condition. This data fits our hypotheses and also demonstrates that EMG can be used very effectively to validate the success of response-related experimental manipulations, for example, to validate assumptions about the presence and amount of response conflict in experimental paradigms.^2^

Importantly, in line with our expectations stimulus analysis was maintained in both the effectively and neutrally cued conditions. First, significant numerical distance effects appeared in RT in both cueing conditions with no interaction with cue. The presence of numerical distance effects provides evidence that a refined analysis of number magnitude happened in both the effectively and neutrally cued conditions [44]. Second, physical size distance effects also appeared in RT in both cueing conditions with no interaction with cue. These effects suggest that not only the task-relevant but also the task-irrelevant stimulus dimension was processed in the effectively cued condition at the same level as in the neutrally cued condition. Third, both RT and P300 congruency effects were present in both cueing conditions. This again suggests that the task-irrelevant stimulus dimension had an effect on stimulus processing in both conditions. All the above observations indicate that the effectively cued condition did not become a simple Go/No-Go task where stimulus conflict could not be detected on an a priori basis. In contrast, the data suggest that stimulus analysis was well maintained in the effectively cued condition.

Behavioral data were in agreement with EMG data. Congruency effects were somewhat stronger in the neutrally cued condition than in the effectively cued condition. First, while RT congruency effects were significant in both neutrally and effectively cued trials, the neutral vs. incongruent and the congruent vs. incongruent reaction time differences were significantly larger in the neutrally cued than in the effectively cued condition. Second, while there was no congruency effect in accuracy in the effectively cued trials, there was strong accuracy congruency effect in neutrally cued trials. The above two findings show that congruency effects were stronger in the effectively than in the neutrally cued condition. EMG data offers an explanation for these stronger behavioural congruency effects: there was much larger response conflict in the neutrally cued condition than in the effectively cued condition. At the same time, as shown in the above paragraph, perceptual analysis of stimuli happened at similar levels in both conditions. The above suggests that our hypothesis was correct; only or primarily only stimulus conflict contributed to congruency effects in the effectively cued condition. In contrast, both stimulus and response conflict contributed to congruency effects in the neutral cue condition. It is interesting to speculate whether the size of the incongruent vs. neutral and the congruent vs. incongruent difference values in the neutrally cued condition can characterize the amount of stimulus + response conflict. Were this true, it could be assumed that this overall conflict decreased by 21% in the neutral vs. incongruent contrast and by 24% in the incongruent vs. congruent contrast. The question follows whether these proportions reflect the contribution of response conflict to overall conflict effects in the Stroop paradigm user here.

### The N450 effect and response conflict

Similar to previous studies, the N450 ERP effect appeared with centro-parietal topography and negative polarity in incongruent minus congruent and incongruent minus neutral difference potentials [3-6,24,25]. As the N450 has now been replicated several times in Stroop paradigms it can indeed be considered a robust marker of conflict processing in ERP studies. It is to note, the consistent appearance of the N450 in Stroop studies excludes that it could be related to some specific aspect of our task (e.g. the Go/NoGo requirement). Most importantly, according to our data the N450 appeared with similar overall timing, peak latency, topography and polarity both when there was response conflict (neutral cue condition) and when there was no or at least highly decreased response conflict (effective cue condition). In addition, the N450 effect appeared only in stimulus-locked but not in response-locked trials. This also suggests that the N450 effect is more related to stimulus than to response processing. Further, there were no Cue × Congruency interactions during the time interval of the appearance of the N450 even when the statistical threshold was decreased to control for potential Tpye-II errors. The above demonstrates that the N450 was sensitive to stimulus conflict rather than to response conflict. The outcome further highlights the methodological advantage of using EMG for checking the validity of the response conflict manipulation. In the absence of EMG data it could be argued that there were no Cue × Congruency interactions in ERPs because the response conflict manipulation was not successful. However, here EMG provides positive evidence for the success of the response conflict manipulation which makes the ERP data clear to interpret. Hence, the combination of EEG and EMG provides a significantly more robust interpretative framework than using only behavioral paradigm-level dissociation of stimulus and response conflict.

The robust nature of the N450 effect in ERP studies is reminiscent of the robust nature of ACC conflict effects in fMRI studies. Can the N450 ERP effect and ACC fMRI effects reflect the activity of the same neural circuits involved in conflict processing? This question is highly relevant because it may become possible to time fMRI ACC conflict effects by using the N450. In relation to this question, previous ERP source localization results pointed to the ACC as a possible source of the N450 effect [3,6,26]. However, because of the inverse problem of EEG the accuracy of source localization results is unknown. Hence, it is likely, but cannot be held for certain at this point, that the N450 reflects ACC conflict effects. A potential resolution of the problem may be provided by future combined EEG/fMRI studies which could constrain EEG source localization results with co-recorded fMRI data. This technique may provide better source estimation results than pure EEG studies. Further, it is also possible that while the N450 reflects ACC conflict effects it is not sensitive to all ACC effects. In fact, our data seems to support this assumption. Specifically, it has been shown that the ACC can be engaged by both stimulus and response conflict [2]. However, ACC activations related to the two kinds of conflict were anatomically non-overlapping. Stimulus conflict affected an ACC area which was more posterior and dorsal than the ACC area involved in response conflict. Hence, it is a likely possibility that the N450 is related to more dorsal stimulus-conflict effects but not to more anteriorly localized response-conflict effects. This hypothesis is in line with our recent source localization results finding a dipole solution for the N450 in the dorsal ACC [6]. For example, ERPs may not be sensitive to response conflict-related ACC effects because the appropriate brain sources may not be optimally aligned for detection with EEG. A related question is whether the N450 is associated with the fronto-central N2 conflict effects, frequently related to ACC activity, appearing in flanker [45,46] and GoNo-Go tasks [47].

Finally, in line with the data from our current study there is now accumulating evidence that the N450 ERP effect is indeed not related to response conflict. First, in our previous two studies we have sorted the data into quickly and slowly responded trials [25,29]. In both studies, slowly responded trials showed substantially larger response conflict than quickly responded trials. However, the N450 appeared with equal amplitude in both quickly and slowly responded trials. This pattern of results supports that the N450 was not modulated by the amount of response conflict. (However, unlike the current study, these previous studies did not explicitly manipulate response conflict.) Importantly, the presence of the N450 in both slowly and quickly responded trials excludes that the N450 would be a correlate of task-difficulty. Were this the case, the N450 should have differed substantially across quickly/slowly responded trials. However, this was not the case. In addition to the above, another study used a color matching paradigm which is similar to a Stroop task [48]. An N450-like amplitude modulation appeared in this task as well, contrary to the fact that the paradigm did not include response conflict in principle Similar N450 results were reported by Mager et al. [49] in another Stroop study which also excluded response conflict. However, in these studies there was no direct measure of response conflict. In summary, all the above studies are in excellent agreement with the data from our current study where we explicitly manipulated and measured response conflict. Hence, it is a reasonable conclusion that the N450 effect indexes either stimulus conflict or a more general abstract level of conflict, rather than response conflict.

### Conflict-related ERP effects besides the N450

The P300 peak latency data is in-line with our above conclusion that ERPs mainly reflect stimulus conflict effects: Similar to our previous numerical Stroop studies, there was a congruency effect on the peak latency of the P300 ERP wave [6,27]. As the peak latency of the P300 is usually thought to be related to stimulus analysis [37], such data can be considered to reflect stimulus conflict effects. Interestingly, consistent P300 congruency effects in the numerical Stroop paradigm are in sharp contrast with the lack of such effects in the classical color-word Stroop paradigm [35,36]. Recently, we have also examined P300 effects in the classical color-word Stroop paradigm and found no congruency effects on P300 latency [29]. A possible explanation for this discrepancy is that numerical meaning and the physical size of digits may be more salient or more directly interpretable stimulus dimensions than word meaning and therefore they may result in larger stimulus conflict than word-reading effects [6]. This stronger stimulus conflict could then show up in P300 latency in the numerical but not in the color-word Stroop paradigm. It is important to note that P300 latency did not show any cue effects or Cue × Congruency interactions. It is also important to note that N450 effects appear on top of the P300 wave. However, due to the volume conducted nature of EEG signals it is not possible to tell whether N450 effects are related to the modulations of the P300, or represent an entirely independent psycho-physiological phenomenon. This is an interesting avenue for further research. There were no effects on the latency of P1 and N2 ERP peaks. Therefore, latency shifts of early ERP waves could not affect later congruency effects.

Besides the N450 effect, there was a second congruency effect between 560-660 ms. This effect had positive polarity in incongruent minus congruent and in incongruent minus neutral difference potentials and appeared over parietal electrodes. Such an effect has been described in previous studies but has not yet been clearly interpreted [3,4,24]. A simple explanation would be that this later congruency effect is an amplitude modulation of the centro-parietal P3b wave which is known to be sensitive to strategical expectations of participants [50]. The only Cue × Congruency interaction in ERP amplitude was detected between 230-280 ms only when setting a lower statistical threshold than the one used for detecting congruency effects. This brief effect temporally preceded the N450 but did not overlap with it and had different scalp distribution than the N450. Hence, the N450 and the interaction effect seem to be independent from each other. Currently we cannot speculate about the anatomical source of the interaction effect.

## Conclusion

In a cued numerical Stroop paradigm we have shown that the N450 ERP effect, a robust marker of Stroop conflict, is not related to response conflict processing. Rather, the data suggest that the N450 is related to stimulus conflict, or to an abstract level of conflict processing. The consistency of its appearance and previous source localization results make it likely that the N450 can be related to the conflict processing activity of the ACC. Therefore, the N450 can potentially be used to time conflict-related ACC activity. Joint EEG/fMRI studies could test the above hypothesis. Methodologically we have shown that EMG can be used successfully to validate the presence/absence of response conflict independently of paradigmatic assumptions. Therefore, EMG can be a valuable tool in fMRI/EEG conflict studies.

## End Notes

^1 ^It is to note that while positive EMG findings can confirm the presence of response conflict with certainty, the lack of EMG findings does not allow to conclude that there is no response conflict.

^2 ^It is to note that neither the EMG, a peripheral measure of response activation, nor the LRP, a central measure of response activation, was able to detect incorrect response activation in the effectively cued condition. Hence, EMG and LRP data suggest that there was indeed no (or undetectable) incorrect response activation in the effectively cued condition at both the central and peripheral levels. As outlined above, the crucial result is not the lack of incorrect response activation in the effectively cued condition. Rather, the crucial result is that there was a highly significant difference in incorrect response activation between the effectively and neutrally cued conditions.

## Competing interests

The authors declare that they have no competing interests.

## Authors' contributions

DS designed the study, analyzed the data and wrote the manuscript. FS contributed to design and programmed the experimental paradigm. Both authors read and approved the final manuscript.

## Supplementary Material

Additional file 1 **Figure S1**. The numerical Stroop task. (A) Example stimuli. Participants press a button on the side where they see the numerically larger number. (B) Processing model of the task: The numerical and physical dimensions of the stimulus are analyzed in parallel resulting in numerical magnitude and physical size stimulus representations ('Repr.'). Stimulus conflict can appear when the evaluation of numerical/physical size representations differ. Further, the parallel processed stimulus representations may than trigger the parallel activation of correct and incorrect response channels, potentially resulting in response conflict. Usually only the correct response channel will result in an overt behavioral response. Electro-myography can provide a direct measure of both correct and incorrect response channel activation. Click here for file

Additional file 2 **Figure S2**. Electrode positions and labels in the 128-channel hydro-cell electrode net. The reference electrode (vertex) is electrode 129. Click here for file

Additional file 3 **Figure S3**. EMG signal in the correct response hand in the effectively (Eff-C) and neutrally cued (Neut-C) conditions. Cong = Congruent. Incon = Incongruent. Click here for file

Additional file 4 **Figure S4**. ERPs split by congruency conditions. (A) Global field power (GFP). (B) ERPs averaged for centro-parietal electrodes 129, 31, 55, 80, 54, 55, 79, 61, 62 and 78. The time course of congruency effects detected by ANOVAs are shown by thick horizontal lines. Congr = Congruent. Incongr = Incongruent. Click here for file

Additional file 5 **Figure S5**. The topography of Cue main effects (p < 0.025) in ERPs. Topographies are shown for [effectively cued condition] minus [neutrally cued condition] difference potentials. Click here for file

Additional file 6 **Figure S6**. The topography of Cue × Congruency interactions (p < 0.025) in ERPs in the effectively (Eff-C) and neutrally cued (Neut-C) conditions. The amplitude of ERPs in the neutral condition is shown on the left for reference. The amplitude of difference topographies is shown on the right (Congr: Cognruent; Incongr: Incognruent). The upper row shows the effectively cued condition, the lower row shows the neutrally cued condition. Click here for file

Additional file 7 **Figure S7**. The Lateralized Readiness Potential in the cued (A) and non-cued (B) conditions. Click here for file

## References

[B1] Liston C Matalon S Hare TA Davidson MC Casey BJ Anterior cingulate and posterior parietal cortices are sensitive to dissociable forms of conflict in a task-switching paradigm Neuron 2006 50 643 653 10.1016/j.neuron.2006.04.015 16701213

[B2] Van Veen V Carter SC Separating semantic conflict and response conflict in the stroop task: a functional fMRI study Neuroimage 2005 27 497 504 10.1016/j.neuroimage.2005.04.042 15964208

[B3] Liotti M Woldorff MG Perez R Mayberg HS An ERP study of the temporal course of the Stroop color-word interference effect Neuropsychologia 2000 38 701 711 10.1016/S0028-3932(99)00106-2 10689046

[B4] Szucs D Soltesz F Event-related potentials dissociate facilitation and interference effects in the numerical Stroop paradigm Neuropsychologia 2007 45 3190 3202 10.1016/j.neuropsychologia.2007.06.013 17675108

[B5] West R Neural correlates of cognitive control and conflict detection in the Stroop and digit location tasks Neuropsychologia 2003 41 1122 1135 10.1016/S0028-3932(02)00297-X 12667546

[B6] Szucs D Soltesz F White S Motor conflict in Stroop tasks: direct evidence from single-trial electro-myography and electro-encephalography Neuroimage 2009 47 1960 1973 10.1016/j.neuroimage.2009.05.048 19481157

[B7] Hock HS Egeth H Verbal interference with encoding in a perceptual classification task J Exp Psychol 1970 83 299 303 5480902 10.1037/h0028512

[B8] Morton J Chambers SM Selective attention to words and colours Q J Exp Psychol 1973 25 387 397 10.1080/14640747308400360

[B9] Posner MI Snyder CRR Solso RL Attention and cognitive control Information processing and cognition: the Loyola symposium Vol 1975 Hillsdale, NJ: Erlbaum 55 85

[B10] Botvinick M Cohen JD Carter CS Conflict monitoring and anterior congulate cortex: an update Trends Cogn Sci 2004 8 539 546 10.1016/j.tics.2004.10.003 15556023

[B11] Botvinick M Cohen JD Carter CS Braver TS Barch DM Conflict monitoring and cognitive control Psychological Review 2001 108 624 652 10.1037/0033-295x.108.3.624 11488380

[B12] Botvinick M Nystrom LE Fissel K Carter SC Cohen JD Conflict monitoring versus selection for action in anterior cingulate cortex Nature 1999 402 179 181 10.1038/46035 10647008

[B13] Cohen JD Botvinick M Carter CS Anterior cingulate and prefrontal cortex: who's in control? Nat Neurosci 2000 3 421 423 10.1038/74783 10769376

[B14] Van Veen V Cohen JD Botvinick MM Stenger VA Carter S Anterior cingulate cortex, conflict monitoring and levels of processing Neuroimage 2001 14 1302 1308 10.1006/nimg.2001.0923 11707086

[B15] Badre D Wagner AD Selection, integration, and conflict monitoring: assessing the nature and generality of prefrontal cognitive control mechanisms Neuron 2004 41 473 487 10.1016/S0896-6273(03)00851-1 14766185

[B16] Melcher T Gruber O Decomposing interference during stroop performance into different conflict factors: an event-related fMRI study Cortex 2009 45 189 200 10.1016/j.cortex.2007.06.004 19150520

[B17] Weissman DH Giesbrecht B Song AW Mangun GR Woldorff MG Conflict monitoring in the human anterior cingulate cortex during selective attention to global and local object features Neuroimage 2003 19 1361 1368 10.1016/S1053-8119(03)00167-8 12948694

[B18] Weissman DH Gopalakrishnan A Hazlett CJ Woldorff MG Dorsal anterior cingulate cortex resolves conflict from distracting stimuli by boosting attention toward relevant events Cereb Cortex 2005 15 229 237 15238434 10.1093/cercor/bhh125

[B19] Egner T Delano M Hirsch J Speparate conflict-specific cognitive control mechanisms in the human brain Neuroimage 2007 35 940 948 10.1016/j.neuroimage.2006.11.061 17276088

[B20] Kerns JG Anterior cingulate and prefrontal cortex activity in an FMRI study of trial-to-trial adjustments on the Simon task Neuroimage 2006 33 399 405 10.1016/j.neuroimage.2006.06.012 16876434

[B21] Szucs D Soltesz F The interaction of task-relevant and task-irrelevant stimulus features in the number/size congruency paradigm: an ERP study Brain Res 2008 1190 143 158 18076868 10.1016/j.brainres.2007.11.010

[B22] Aine CJ Harter MR Hemipsheric differences in event-related potentials to Stroop stimuli Ann N Y Acad Sci 1984 425 154 156 10.1111/j.1749-6632.1984.tb23525.x 6588822

[B23] Atkinson CM Drysdale KAD Fulham WR Event-related potentials to Stroop and reverse Stroop stimuli Int J Psychophysiol 2003 47 1 21 10.1016/S0167-8760(02)00038-7 12543443

[B24] Appelbaum LG Meyerhoff KL Woldorff MG Priming and backward influences in the human brain: processing interactions during the stroop interference effect Cereb Cortex 2009 19 2508 21 10.1093/cercor/bhp036 19321654 PMC2764508

[B25] Szucs D Soltesz F Bryce D Whitebread D Real-time tracking of motor response activation and response competition in a stroop task in young children: a lateralized readiness potential study J Cogn Neurosci 2009 11 2195 2206 19296726 10.1162/jocn.2009.21220

[B26] Hanslmayr S Pastotter B Bauml KH Gruber S Wimber M Klimesch W The electrophysiological dynamics of interference during the stroop task J Cogn Neurosci 2008 20 215 225 10.1162/jocn.2008.20020 18275330

[B27] Szucs D Soltesz F Jarmi E Csepe V The speed of magnitude processing and executive functions in controlled and automatic number comparison in children: an electro-encephalographic study Behavioral Brain Functions 2007 3 23 10.1186/1744-9081-3-23 PMC1872027 17470279

[B28] Gratton G Coles MGH Sirevaag EJ Eriksen CW Donchin E Pre- and poststimulus activation of response channels: a psychophysiological analysis J Exp Psychol Hum Percept Perform 1988 14 331 344 2971764 10.1037//0096-1523.14.3.331

[B29] Szucs D Soltesz F Stimulus and response conflict in the color-word Stroop task: a combined electro-myography and event-related potentials study Brain Research 2010 1325 63 76 20153298 10.1016/j.brainres.2010.02.011

[B30] Burle B Roger C Allain S Vidal F Hasbroucq T Error negativity does not reflect conflict: a reappraisal of conflict monitoring and anterior cingulate activity J Cognitive Neurosci 2008 20 1 19 10.1162/jocn.2008.20013 18345992

[B31] Coles MGH Gratton G Bashore TR Eriksen CW Donchin E A psychophysiological investigation of the continuous flow model of human information processing J Exp Psychol: Human Percept Perform 1985 11 529 553 2932529 10.1037//0096-1523.11.5.529

[B32] Eriksen CW Coles MG Morris LR O'Hara WP An electromyographic examination of response competition Bull Psychonomic Soc 1985 23 165 168

[B33] Masaki H Takasawa N Yamazaki K An electrophysiological study of the locus of the interference effect in a stimulus-response compatibility paradigm Psychophysiology 2007 37 464 472 10934905

[B34] Henik A Tzelgov J Is three greater than five: the relation between physical and semantic size in comparison tasks Mem Cogn 1982 10 389 395 10.3758/BF03202431 7132716

[B35] Duncan-Johnson CC Koppel BS The Stroop effect: brain potentials localize the source of interference Science 1981 21 938 940 7302571 10.1126/science.7302571

[B36] Ilan AB Polich J P300 and response time from a manual Stroop task Clin Neurophysiolology 1999 110 367 373 10.1016/S0168-5597(98)00053-7 10210626

[B37] Kutas M McCarthy G Donchin E Augmenting mental chronometry: the P300 as a measure of stimulus evaluation in time Science 1977 197 792 795 10.1126/science.887923 887923

[B38] Falkenstein M Hohnsbein J Hoormann J Effects of choice complexity on different subcomponents of the late positive complex of the event-related potential Electroencephalogr Clin Neurophysiol 1994 92 148 160 10.1016/0168-5597(94)90055-8 7511512

[B39] Verleger R On the utility of P3 latency as an index of mental chronometry Psyhophysiology 1997 34 131 156 10.1111/j.1469-8986.1997.tb02125.x 9090263

[B40] Fridlund AJ Cacioppo JT Guidelines for human electromyographic research Psychophysiol 1986 23 567 589 10.1111/j.1469-8986.1986.tb00676.x 3809364

[B41] Lehman GJ McGill SM The importance of normalization in the interpretation of surface electromyography: A proof of principle J Manipulative Physiol Ther 1999 22 444 446 10.1016/S0161-4754(99)70032-1 10519560

[B42] Lehmann D Skrandies W Reference-free identification of components of checkerboard evoked multichannel potential fields Electroencephalography Neurophysiology 1980 48 609 621 10.1016/0013-4694(80)90419-8 6155251

[B43] Skrandies W Visual information processing: topography of brain electrical activity Biol Psychol 1995 10 1 15 7647172 10.1016/0301-0511(95)05111-2

[B44] Tzelgov J Meyer J Henik A Automatic and intentional processing of numerical information J Exp Psychol Learn Mem Cogn 1992 18 166 179

[B45] Bartholow BD Pearson MA Dickter CL Sher KJ Fabiani M Gratton G Strategic control and medial frontal negativity: beyond errors and response conflict Psychophysiol 2005 42 33 42 10.1111/j.1469-8986.2005.00258.x 15720579

[B46] Kopp B Rist F Mattler U N200 in the flanker task as a neurobehavioral tool for investigating executive control Psychophysiology 1996 33 282 294 10.1111/j.1469-8986.1996.tb00425.x 8936397

[B47] Nieuwenhuis S Yeung N Van Den Wildenberg W Ridderinkhof KR Electrophysiological correlates of anterior cingulate function in a go/no-go task: effects of response conflict and trial type frequency Cogn Affect Behav Neuroscience 2003 3 17 26 10.3758/CABN.3.1.17 12822595

[B48] Mager R Meuth SG Krauchi K Schmidlin M Muller-Spahn F Falkenstein M Mismatch and conflict: neurophysiological and behavioral evidence for conflict priming J Cogn Neurosci 2009 21 2185 2194 10.1162/jocn.2008.21154 18855548

[B49] Mager R Bullinger AH Brand S Schmidlin M Schärli H Müller-Spahn F Störmer R Falkenstein M Age-related changes in cognitive conflict processing: an event-related potential study Neurobiol Aging 2007 28 1925 1935 10.1016/j.neurobiolaging.2006.08.001 16973245

[B50] Donchin E Presidential address, 1980. Suprise!...Suprise? Psychophysiol 1981 18 493 513 10.1111/j.1469-8986.1981.tb01815.x 7280146

